# Progesterone Receptor Expression in the Benign Prostatic Hyperplasia and Prostate Cancer Tissues, Relation with Transcription, Growth Factors, Hormone Reception and Components of the AKT/mTOR Signaling Pathway

**DOI:** 10.31557/APJCP.2020.21.2.423

**Published:** 2020

**Authors:** Liudmila V Spirina, Irina V Kovaleva, Evgeny A. Usynin, Alexey K Gorbunov, Irina V Kondakova

**Affiliations:** 1 *Leader Researcher, Laboratory of Tumor Biochemistry, *; 4 * Department of Surgical, Cancer Research Institute, Tomsk National Research Medical Center, Russian Academy of Medical Sciences, *; 2 *Division of Biochemistry and Molecular Biology with Course of Clinical Laboratory Diagnostic, *; 3 *Student of Medico-Biological Faculty, Siberian State Medical University, Russian Federation. *

**Keywords:** Progesterone receptor (PR), benign prostatic hyperplasia, prostate cancer, transcription and growth factors

## Abstract

**Background:**

Progesterone receptor (PR) is a critical regulator in reproductive tissues that controls a variety of cellular processes. The objective of the study was to study the PR expression in patients with benign prostatic hyperplasia and prostate cancers in connection with the transcription, growth factors, AR, ERα, ERβ, and components of the AKT/mTOR signaling pathway expression.

**Materials and methods:**

Ninety-seven patients with prostate pathology were enrolled in the study. Forty-two patients had benign prostatic hyperplasia (BH). Fifty-five patients had locally advanced prostate cancer (PCa). The PSA level and the amount of testosterone in the serum were measured using an ELISA assay. The expression level of NF-κB p65, NF-κB p50, HIF-1, HIF-2, growth factor VEGF, VEGFR2, CAIX, as well as AR, ERα, ERβ, PR, Brn-3α, TRIM16 were quantified by RT-PCR. The protein level of Brn-3α, TRIM16 was detected by Western Blotting.

**Results:**

Growth in PR expression was observed in PCa tissues compared to BH ones without changes in the clinical and pathological features of the patients. An increase in PR expression was detected in patients with PCa compared to BH. Its mRNA level depended on the expression of AR, Brn-3α, and TRIM16, components of the AKT/mTOR signaling pathway, transcription, and growth factors. An increase in the TRIM16 expression in the PCa tissues was noted in the case of a low PR level. We revealed the growth in PR expression was accompanied by the suppression of the signaling cascade activity, AR, Brn-3α mRNA level, and the enhanced PTEN expression in PCa tissues. The increase in PR expression in PCa led to a decrease in the level of mRNA of NF-κB, HIF-1, VEGF, and VEGFR2.

**Conclusion:**

In general, the data indicated the significance of the PR expression in the development of the prostate pathology that affected the cross-talk between the steroid hormone reception and signal transduction.

## Introduction

Prostate cancers (PCa) are in second place according to tumor incidence and the fifth leading cause of cancer-related deaths in men. Besides, it is the most frequent reason for mortality among men (Rebbeck TR, 2017). It is known its sickness rate increases every year. 27046 new cases of prostate cancer were identified in 2012. So, the cancer prevalence is 40.2 per 100 thousand men. The average increase in morbidity is 9.83%, and it takes the first place according to the rate of cancer prevalence (Heidenreich et al., 2014)

The prostate cancer (PCa) development is closely associated with the impact of the steroid hormones, represented by gonadal steroid hormones and corticosteroids, which regulate the main vital processes (Taplin and Balk, 2004). Steroid hormone receptors belong to a large family of intracellular receptors, which are activated by the action of specific ligands. Testosterone, dihydrotestosterone and androgen receptor (AR) plays a crucial role in the hormonal carcinogenesis (Quayle et al., 2007)

However, researchers are increasingly attracted by PRs and their ligand - progesterone [4], which is a precursor to the synthesis of testosterone, cortisol. There are two progesterone receptor isoforms (PGRA, PGRB) in PCa tissue (Grindstad et al., 2015). It is believed that the level of PGRB expression is associated with a poor prognosis of the disease and an inadequate response to ADT (Grindstad et al., 2018; Thomas et al., 2014). The canonical pathway of progesterone activation occurs as follows: when PR is bound to its ligand, it penetrates the nucleus. It connects with the PRE (progesterone responsible element), which is a genomic effect. Besides, PR can also have non-genomic effects, which is associated with the activation of non-nuclear mPRs receptors (membrane progesterone receptors) and PGRMC1 (progesterone receptor membrane component), affecting cell proliferation and the development of invasive potential along the non-canonical path (Thomas et al., 2014).

AR and estrogen (ER) are implicated to have essential functions, whereas the progesterone receptor remains largely under-investigated despite the high sequence and structural similarities between PR and AR. ER has two subtypes: ERα and ERβ. Each of them is encoded in the genome. There is data about the preferential expression of one of the receptor subtypes in various tissues (Mohammed et al., 2015). The ER/PR axis is crucial in PCa, where the physiological outcome would be affected by the differential signaling initiated by the canonical and the non-canonical receptors (Sen et al., 2017). The crosstalk between the ER/PR axis and the growth factor/PI3K/AKT/mTOR system is also highly relevant. 

Progesterone, being a source of de novo androgen synthesis, can activate mutant AR (Chen et al., 2017). PR is also involved in a large number of alternative, non-genomic signaling cascades, e.g., PR can activate MAPK and PI3K/AKT pathways, which leads to the regulation of gene expression (Lee et al., 2016). 

The cross-talk between PR and growth factors results in progesterone-independent activation of PR. Transcriptional activity of PR, as well as its dynamic impact on processes such as cell migration and adhesion, are crucial for PCa progression. There is evidence that PR can act as an oncosuppressor, reducing the prostate cells malignization (Piasecka et al., 2015). Recent progress in our understanding of PR function suggests that this receptor may exert an inhibitory effect on benign prostatic hyperplasia (BH) and PCa progression (Chen et al., 2017).

Currently, it is believed that the effect of steroids may be associated with a complex of other nuclear peptides. The transcription factor Brn-3α can affect the transcriptional activity of androgen and estrogen receptors (Diss et al., 2006). It is observed the participation of TRIM16 protein in the pathogenesis of hormone-dependent tumors due to its “anti-estrogenic effect” (Qi et al., 2016). Additionally, they are involved in the intracellular processes, such as proliferation, cell differentiation, and programmed death - apoptosis (Diss et al., 2006; Qi et al., 2016)

The transcription factor NF-κB is a critical determinant of prostate cancer biology, which is implicated in cancer development (Jin et al., 2016). Hypoxia is a reduction in the normal concentration of tissue oxygen, which occurs in prostate cancer. It is known a crucial effect of hypoxia is the induction of genetic alterations and angiogenic stimulation, leading to a more aggressive cell phenotype and malignant progression (Grivas et al., 2016; Ambrosio et al., 2016). It increases hypoxia-induced factor (HIF) level, HIF-dependent proteins endothelial growth factor (VEGF), carbonic anhydrase IX (CAIX), and VEGFR2 receptor (Stifter and Dordevic, 2014; Rivera-Pérez et al., 2018). Recent advances have explored the central role of HIF-1α in PCa development and progression. The rate of HIF-1α protein expression in PCa was significantly higher than in nonmalignant prostate tissues and associated with clinicopathological significance for patients suffering from PCa (Huang et al., 2018).

Our earlier studies have shown that an increase in ERα expression is associated with the formation of a malignant tumor, in contrast to AR. A high level of AR is specific for both PCa and benign prostatic hyperplasia (BH) (Spirina et al., 2018). Besides, the PCa progression is followed by the transcription factors Brn-3α and TRIM16 level change (Diss et al., 2006; Qi et al., 2016; Spirina et al., 2018). Activation of the AKT/mTOR signaling pathway in the PCa tissue was accompanied by enhanced PTEN expression (Spirina et al., 2018).

Molecular mechanisms of PR action are complex and diverse, which makes the possibility of studying them most attractive in the light of the search for new markers of prognosis and the course of the disease. In this regard, the purpose of the study was to study the expression of PR in the tissue of patients with BH and PCa in connection with the transcription, growth factors, AR, ERα, ERβ, and components of the AKT/mTOR signaling pathway expression.

## Material and Methods

A total group of 97 patients with PCa was enrolled in the investigation. The age of patients was from 40 to 80 years. Patients had a verified diagnosis of PCa T2-3N0-1M0-1. Fifty-five patients with localized and locally advanced prostate cancer were included in the investigation. The control group consists of forty-two men with BH. 

All patients admitted to the Hospital at the Cancer Research Institute, Tomsk National Research Center, Russian Academy of Medical Sciences, Tomsk, Russian Federation. The study was approved by the Local Committee for Medical Ethics, and all patients provided written informed consent. Tumor tissue samples, hyperplastic, and histologically normal tissue samples were used for investigation. Specimens were reviewed separately by two independent pathologists. Tissues were taken from the transrectal prostate biopsy and immediately frozen. Normal samples were procured from patients with bladder cancer undergoing cystectomy.

The Local Committee approved the study for Medical Ethics, and all patients signed the written informed consent. The research was carried out in concordance with the Declaration of Helsinki (2008) of the World Medical Association. 

Determination of PSA and testosterone levels. The level of PSA and testosterone were determined by ELISA assay. 

*RNA extraction*

The postoperative tumor samples were incubated in RNAlater solution (Ambion, USA) for 24-hours at + 4°C and then stored at -80°C. Total RNA was extracted using the RNeasy Mini Kit (Qiagen).

RT-qPCR. PCR was conducted in 25 μl reaction volumes containing 12.5 μl BioMaster HS-qPCR SYBR Blue (2X) (“Biolabmix” Russia) and 300 nanoM of each primer. Primers were selected using the Vector NTI Advance 11.5 program and the NCBI database (http: //www.ncbi.nlm. nih.gov/nuccore) (Table 1).

A pre-incubation at 95°C for 10 min was to activate the Hot Start DNA polymerase and denature DNA and was followed by 45 amplification cycles of 95°C denaturation at 95 0 for 10 sec, 60°C annealing at 60 0 for 20 sec (iCycler iQ™, BioRad).

The fold changes were calculated by the ΔΔCt method (the total ΔΔCt = fold of cancerous/normal tissue gene level), using normal tissue. A ratio of specific mRNA/GADPH (GADPH as a respective control) amplification was then calculated. The relative gene expression was defined as Relative Units (RLU).

Preparing tissue homogenates. Tissue samples (100 mg) were homogenized and then resuspended in 300 μL of 50 mM Tris-HCl buffer (pH=7.5) containing 2 mM ATP, 5 mM MgCl2, 1 mM dithiothreitol, 1 mM EDTA, and 100 mM NaCl. The homogenate was centrifuged at 10,000×g for 60 minutes at 4°C.

*Determination of expression levels of AKT/m-TOR signaling pathway components*

*Electrophoresis. SDS-PAGE (Laemmli) was used*

Western Blot Analysis. The protein was transferred to a 0.2-/xm pore-sized PVDF membrane (GE Healthcare, UK), either at 150 mA or 100 V for one h by using a Bio-Rad Mini Trans-Blot electrophoresis cell. The membrane was incubated in a 1:2500 dilution of monoclonal mouse Brn-3α (Abcam, USA) and TRIM16 (Thermo Fisher Scientific, USA) at four ºС overnight. PVDF samples were incubated in Amersham ECL Western Blotting Detection Analysis System (Amersham, USA). The results were standardized using the beta-actin expression in a sample and were expressed in percentages to the protein content in non-transformed tissues. The level of protein in normal non-altered tissue was indicated as 100%.

Statistical analysis. Statistical analysis was performed using SPSS 19.0 software. Data are expressed as median (interquartile ranges). The non-parametric Mann-Whitney U test was used.

## Results

**Figure 1:**
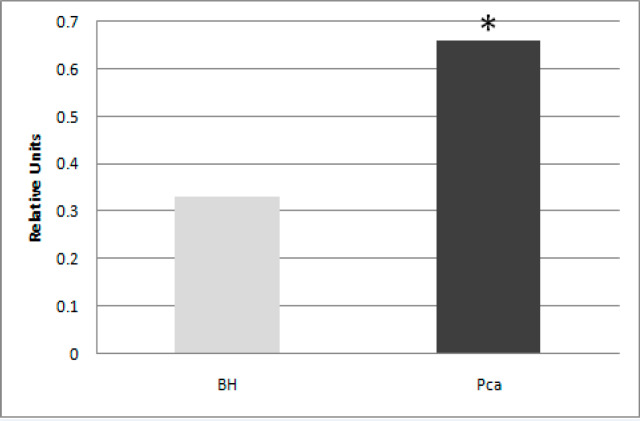
Progesterone Receptor (PR) Expression in BH and PCa Tissues. Note: PR expression was enhanced in PCa tissues compared to BH ones. The significance of PR in prostate cells remains unknown. It is shown PR is involved in a large number of alternative, non-genomic signaling cascades, growth factors activation that results in cancer biology change

Clinical and morphological parameters of patients with PCa and BH were shown in Table 2. There was no difference in the age, PSA level, testosterone content, and Gleason score in patients with benign and malignant prostate pathology. Probably, molecular markers and factors associated with them, which can modify the invasive potential of a tumor, may be of great importance for studying the biological features of the prostate gland pathology. In the PCa tissue, it was found an increase in the PR mRNA level by 22.0 times compared to BH (Figure 1). It is known that the PR level, having a variety of genomic and genomic effects, is the most promising indicator. 

In this regard, all patients with PCa were divided into groups: with decreased and increased PR expression (< and > than 1.0 RLU). Twenty-one PCa patients had a PR expression of less than 1.0 RLU (<0.1 RLU). The group of patients with increased, > 1.0 RLU, was composed of 34 PCa patients. Further studies of the expression of AR, ER, Brn-3α, TRIM16, as well as components of the AKT/mTOR signaling pathway, transcription and growth factors were performed in these two groups of patients with PCa.

It was found a prevalence of patients with increased PR expression (> 0.1 RLU) in PCa patients compared to BH (Table 3). The 1.8-fold growth in the percentage of patients with PR expression > 0.1 RLU was detected compared to patients with BH.

It was noted that the growth in the PR expression was not associated with a change in the mRNA level of ERα, ERβ (Table 4). At the same time, a 112.5-fold increase in AR expression was revealed in patients with PCa and PR> 1.0 RLU compared to patients with BH and the same PR expression rate. The TRIM16 expression was shown to be increased by 15.4 times in patients with PCa and PR expression < 1.0 RLU compared to patients with BH and PR rate <0.1 RLU. 

**Figure 2:**
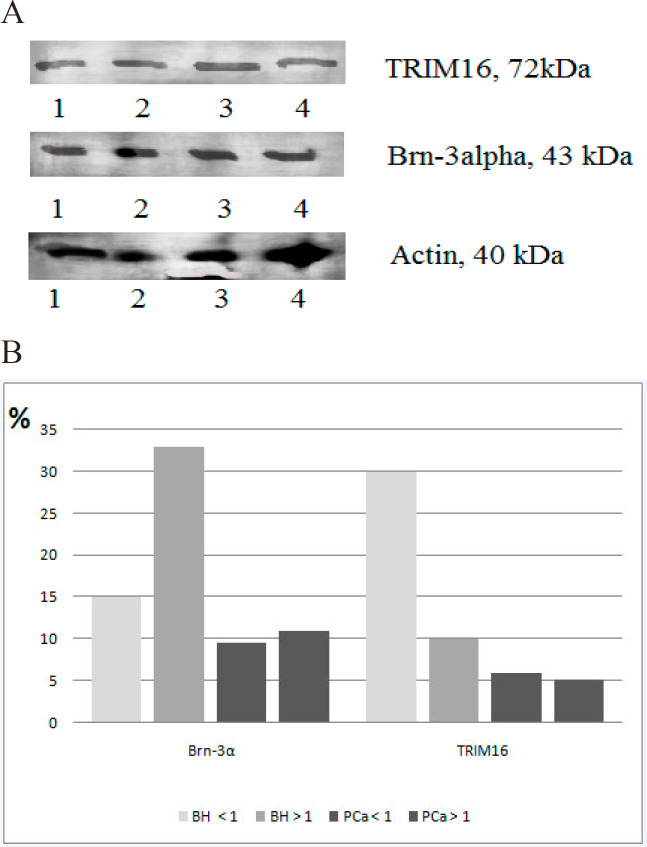
The Protein Level of Nuclear Factors Brn-3α, TRIM16 in BH, and PCa Tissues in Patients with PR Expression <0.01 RLU and >0.01RLU. Note: A - Western blot of transcription factors Brn-3α, TRIM16; 1, 3 - tumor tissue; 2, 4 - normal tissue; B - the content of transcription factors Brn-3α, TRIM16 in the prostate tissue of patients with BH and prostate cancer with PR expression less than 1.0 RLU (<1 RLU.) and higher than 1.0 RLU (> 1 RLU.). Regulatory peptides are shown to be promising factors, which can regulate the transcriptional activity of both AR and ER and can be implicated in hormonal oncogenesis. The revealed data indicated no changes in Brn-3α and TRIM16 protein levels in PCa and BH patients. The significant results were obtained in the study of their mRNA rate

However, in PCa patients, AR expression was reduced by 2.3 times in men with PR expression > 1.0 RLU compared to the group with PR expression < 1.0 RLU. The Brn-3α mRNA level was decreased by 3.6 times in patients with the PR expression > 1.0 RLU compared to patients with PR expression < 1.0 RLU. But there was no change in the protein level of transcription factors Brn-3α and TRIM16 in patients with prostate pathology (Figure 2).

Associations between PR expression and the mRNA level of the AKT/mTOR signaling pathway components, transcription, and growth factors were also studied (Table 5). In PCa patients with PR expression > 1.0 RLU, a 201.1-fold increase in PTEN expression was noted compared to patients with BH with the same PR rate. We revealed the opposite data for transcription factors. A 36.7-fold decrease in the NF-κB p50 expression was observed in patients with PCa and PR mRNA level > 1.0 RLU times compared to patients with BH. Additionally, the NF-κB p50, HIF-1, HIF-2 expression was found decreased in PCa tissues with a PR expression < 1.0 RLU by 4.4; 2.5 and 10 times, respectively, compared to cancers with PR expression > 1.0. We obtained the similar changes in VEGF and its receptor (VEGFR2) expression. Their mRNA levels were decreased by 13.6 and 6.2 times, respectively, in PCa tissues with PR rate > 1.0 RLU compared to cancers with PR <1.0 RLU. 

**Table 1:** The Sequence of Primers

Gene	amplicon	sequence
*CAIX *NM_001216.2	217 bp	F 5¢-GTTGCTGTCTCGCTTGGAA-3¢ R 5¢-CAGGGTGTCAGAGAGGGTGT-3¢
*HIF-1*α NM_001243084.1	188 bp	F 5¢- CAAGAACCTACTGCTAATGCCA-3¢ R 5¢- TTTGGTGAGGCTGTCCGA-3¢
*EPAS1 *NM_001430.4	265 bp	F 5¢- TGGAGTATGAAGAGCAAGCCT-3¢ R 5¢-GGGAACCTGCTCTTGCTGT-3¢
*NFKB1 *NM_001165412.1	144 bp	F 5¢-CGTGTAAACCAAAGCCCTAAA-3¢ R 5¢-AACCAAGAAAGGAAGCCAAGT-3¢
*RELA *NM_001145138.1	271 bp	F 5¢-GGAGCACAGATACCACCAAGA-3¢ R 5¢-GGGTTGTTGTTGGTCTGGAT-3¢
*PTEN *NM_001304717.2	136 bp	F 5¢-GGGAATGGAGGGAATGCT-3¢ R 5¢-CGCAAACAACAAGCAGTGA-3¢
*VEGFA *NM_001025366.2	316 bp	F 5¢-AGGGCAGAATCATCACGAA-3¢ R 5¢-TCTTGCTCTATCTTTCTTTGGTCT-3¢
*KDR *NM_002253.2	306 bp	F 5¢-AACACAGCAGGAATCAGTCA-3¢ R 5¢-GTGGTGTCTGTGTCATCGGA-3¢
*4EBP1 *NM_004095.3	244 bp	F 5¢- CAGCCCTTTCTCCCTCACT -3¢ R 5¢- TTCCCAAGCACATCAACCT -3¢
*AKT1 *NM_001014431.1	181 bp	F 5¢- CGAGGACGCCAAGGAGA -3¢ R 5¢- GTCATCTTGGTCAGGTGGTGT -3¢
*С-RAF *NM_002880.3	152 bp	F 5¢- TGGTGTGTCCTGCTCCCT -3¢ R 5¢- ACTGCCTGCTACCTTACTTCCT -3¢
*GSK3b *NM_001146156.1	267 bp	F 5¢- AGACAAGGACGGCAGCAA -3¢ R 5¢- CTGGAGTAGAAGAAATAACGCAAT -3¢
*70S kinase alpha *NM_001272042.1	244 bp	F 5¢- CAGCACAGCAAATCCTCAGA -3¢ R 5¢- ACACATCTCCCTCTCCACCTT -3¢
*m-TOR *NM_004958.3	160 bp	F 5¢- CCAAAGGCAACAAGCGAT-3¢ R 5¢- TTCACCAAACCGTCTCCAA -3¢
*PDK1* NM_001278549.1	187 bp	F 5¢- TCACCAGGACAGCCAATACA -3¢ R 5¢- CTCCTCGGTCACTCATCTTCA -3¢
*POU4F1 *NM_006237	294 bp	F 5¢- CACGCTCTCGCACAACAA-3¢ R 5¢- ATCCGCTTCTGCTTCTGTCT-3¢
*AR *NM_000044	190 bp	F 5¢- GAGGGACAGCAGGCAGA-3¢ R 5¢- GCTATCAGAACACACACACACACT-3¢
*ESR1 *NM_000125	386 bp	F 5¢- TCCTGATGATTGGTCTCGTCT-3¢ R 5¢- GATGTGGGAGAGGATGAGGA-3¢
*ESR2 *NM_001040275.1	243 bp.	F 5¢- GGTCCATCGCCAGTTATCAC-3¢ R 5¢- GCCTTACATCCTTCACACGA-3¢
*PR *NM_000926	260 bp	F 5-TGCCTATCCTGCCTCTCAAT-3¢ F 5-CTTCCTCCTCCTCCTTTATCTTT-3¢
*TRIM16 *NM_001348119	267 bp	F 5¢- CAATGGAACGGGAAGGAG-3¢ R 5¢- GGACGGTGCTGGCTTCT-3¢
*GAPDH *NM_001256799.2	138 bp	F 5¢- GGAAGTCAGGTGGAGCGA-3¢ R 5¢-GCAACAATATCCACTTTACCAGA-3¢

NM, RNA sequence number in the NCBI Nucleotide Database (http://www.ncbi.nlm.nih.gov/nuccore); F, direct primer; R, reverse primer.

**Table 2:** Clinical and Morphological Data of Patients with Prostate Pathology

Indicators	prostate pathology	
	BH, n=42	PCa, n=55
Age, years	65.0 (61.0; 70.0)	61.0 (57.0; 68.0)
Testosterone, pmol/l	14.3 (11.4; 15.45)	8.6 (4.6; 13.0)
PSA, nmol/l	8.7 (6.1; 17.0)	8.0 (6.0; 12.0)
Gleason score	8.5 (7.0; 9.0)	7.0 (7.0; 9.0)

* - significance of differences compared to BH patients, p<0.05

**Table 3:** The Distribution of the PR Expression < 0.1 RLU and >0.1 RLU in Patients with BH and PCa,% (n)

PR expression, Relative Units	BH	PCa	significance
Decreased (less than 1,0)	70% (n=30)	38% (n=21)	P=0.003
Increased (more than 1,0)	30% (n=42)	72% (n=34)	
ϰ^2^ with Yates correction 9.26			

p, the significance of difference

**Table 4:** AR, ERα, ERβ, Brn-α, TRIM16 Expression in BH and PCa Tissues in Patients with the PR Expression < 0.1 RLU and > 0.1 RLU

Indicative	BH PR expression		PCa PR expression	
	<1.0 RLU	> 1.0 RLU	<1.0 RLU	> 1.0 RLU
AR expression, Relative units	32.0 (8.0; 56.0)	**0.5 (0.00; 4.0)**	**128.00 (16.00; 512.00)**	56.25 (26.00; 256.00)*,#
ERα expression, Relative units	1.0 (1.0; 8.0)	8.0 (2.0; 56.0)	2.00 (0.50; 238.86)	1.87 (0.50; 8.00)
ERβ expression, Relative units	0.02 (0.00; 0.25)	4.0 (0.25; 8.0)	0.25 (0.00; 4.00)	0.13 (0.00; 4.00)
Brn-3α expression, Relative units	3.03 (0.25; 8.00)	1.0 (0.87; 28.0)	**27.76 (11.31; 64.00)**	7.73 (2.14; 16.00)*
TRIM16 expression, Relative units	**0.13 (0.13; 1.0)**	8.0 (0.01; 16.0)	**2.00 (1.00; 16.00)#**	2.00 (0.50; 8.00)

Примечание: *, significance of differences compared to PCa patients with PR expression < 1.0; p<0.05; #, significance of differences compared to BH patients, p<0.05;

**Table 5:** Expression of AKT/m-TOR Components, Transcription and Growth Factors in BH and PCa Tissues in Patients with Decreased and Increased PR Expression

Indicative	BH PR expression		PCa PR expression	
	<1.0 RLU	> 1.0 RLU	<1.0 RLU	> 1.0 RLU
Components of AKT/m-TOR signaling pathway				
PDK	1.0 (1.0; 1.52)	2.0 (1.4; 128.0)	2.00 (1.00; 4.00)	2.00 (1.00; 4.00)
PTEN	128.0 (9.6; 212.0)	**1.0 (1.0;64.0)**	256.00 (64.00; 512.00)	**201.51 (97.01; 512.00)#**
AKT	0.50 (0.06; 0.76)	4.0 (1.0; 64.0)	4.00 (1.00; 7.46)	2.00 (1.00; 4.00)
c-RAF	4.0 (1.0; 8.0)	0.02 (0.01; 8.0)	5.60 (2.14; 16.00)	8.00 (4.00; 16.00)
GSK-3β	0.19 (0.13; 0.25)	4.0 (1.0; 128.0)	0.50 (0.25; 1.00)	0.50 (0.13; 1.00)
mTOR	2.46 (0.09; 2.83)	2.0 (1.0; 56.0)	0.71 (0.62; 2.83)	3.15 (0.71; 5.66)
70s 6 kinase	0.71 (0.04; 1.41)	0.02 (0.01; 8.0)	2.83 (0.71; 5.66)#	2.83 (0.71; 5.66)
4EBP1	0.50 (0.06; 0.76)	0.25 (0.06; 8.0)	1.00 (1.00; 2.00)#	1.00 (0.50; 2.00)
Transcription and growth factors				
NF-κB p65	0.01 (0.00; 0.81)	0.00 (0.00; 12.9)	0.03 (0.03; 0.20)	0.01 (0,00; 0.40)
NF-κB p50	0.69 (0.02; 1.0)	**20.2 (20.2; 128.0)**	**2.41 (0.71; 6.92)**	0.55 (0.04; 1.48)*, #
HIF-1	0.11 (0.05; 6.32)	1.71 (1.71; 54.0)	**0.28 (0.11; 6.54)**	0.11 (0.00; 0.85)*
HIF-2	2.3 (0.5; 6.3)	0.4 (0.4; 28.0)	2.00 (0.13; 11.00)	0.20 (0,00; 1.00)
VEGFR2	0.08 (0.01; 1.0)	0.02 (0.02; 0.07)	**0.32 (0.01; 2.58)**	0.05 (0.01; 0.16)*
VEGF	0.00 (0.00; 1.00)	0.01 (0.01; 28.2)	**0.41 (0.02; 6.82)**	0.03 (0.01; 0.09)*
CA9	0.19 (0.00; 0.33)	0.00 (0.00; 0.63)	0.06 (0.02; 0.09)	0.01 (0.00; 0.14)

*, the significance of differences compared to PCa patients with PR expression < 1.0 RLU; p<0.05; #, the significance of differences compared to BH patients, p<0.05

## Discussion

An increase in PR expression was found in patients with PCa tissues compared to BH ones without changes in clinical and pathological features of the patients (age, PSA level, testosterone content, Gleason score). In previous works, it was shown that a significant indicator of carcinogenesis in the prostate gland was the level of AR (Spirina et al., 2018). But its level didn’t differ in patients with the benign and cancer prostate pathology, remaining elevated in transformed and non-transformed cells. It is known that the associations between the transcriptional activity of AR and PR could affect the disorders of hormone reception and lead to PCa development (Quayle et al., 2007; Chen et al., 2017; Lee et al., 2016). The PR expression was found to be associated with the expression of both AR and its regulatory peptides (Brn-3α and TRIM16) in patients with BH.

Consequently, the increase in the PR level was accompanied by the AR growth in BH tissues. In PCa tissues, we detected the opposite data. The PR expression had a reverse connection with the AR and Brn-3α mRNA levels. The significance of regulatory peptide TRIM16 was revealed in PCa tissues with a low PR level. The growth in TRIM16 expression with a reduced PR mRNA level (< 0.1 RLU) was found to be a sign of prostate cell malignization.

The obtained data have shown the value of PR as a factor involved in the development of the prostate pathology at low AR mRNA rates. A favorable prognostic role of PR for patients with prostate cancer to the response to androgen-deprivation therapy is known (Chen et al., 2017). It is likely to be mediated by the tumor suppressor effect of PR (Chen et al., 2017; Piasecka et al., 2015) and is associated with a decrease in the Brn-3α expression, the primary modulator of the AR, ER transcriptional activity (Spirina et al., 2018).

The PR expression was closely related to the level of the growth factor/PI3K/AKT/mTOR system activation. Lee (2016) revealed the significant connections between the AKT activity, PTEN loss, and PR receptor level (Lee et al., 2016; Spirina et al., 2018). We found that the prostate cancer malignization in high PR expression (> 0.1 RLU) was associated with the rise in PTEN mRNA level, and the decline in NF-κB p50 expression. It is known, NF-κB is a strong oncogenic factor, which is constitutively active in human prostate cancer cells (Jin et al., 2015). Progesterone is the principal growth-inhibitory hormone in the prostatic epithelium and promotes apoptosis (Piasecka et al., 2015). PR may exert an inhibitory effect on BH and PCa progression (Chen et al., 2017). We found that decreased PR expression (< 0.1 RLU) was associated with a low level of growth factors, NF-κB, and HIF expression in PCa. This fact confirms the evidence that PR can act as an oncosuppressor, reducing the prostate cells malignization and cancer progression (Piasecka et al., 2015). 

The transcription factors NF-κB and HIF are implicated in tumorigenesis and are the essential downstream substrates of AKT (Jin et al., 2015; Grivas et al., 2016; Ambrosio et al., 2016). The cross-talk between the studied nuclear factors, components of the AKT/mTOR signaling pathway, and PR can modify the factors affecting the genetic alterations, angiogenesis stimulation. The PCa biology investigation is a promising tool for reducing the aggressiveness of transformed prostate cells and preventing cancer progression. 

In conclusion, an increase in PR expression was detected in patients with PCa compared with BH. Its mRNA level depended on the expression of AR, Brn-3α, and TRIM16, components of the AKT/mTOR signaling pathway, and transcription and growth factors. The association of increased PR expression with a decrease in the AR mRNA, the nuclear factor Brn-3α level, was noted in PCa tissues. An increase in the TRIM16 expression in the PCa tissues was pointed out in the case of a low PR level. The growth in PR expression was accompanied by the suppression of the signaling cascade activity and an enhancing PTEN expression in PCa tissues. The increase in PR expression led to the NF-κB, HIF-1, VEGF, and VEGFR2 overexpression in cancers. In general, the data indicated the significance of the PR expression in the development of the prostate pathology that affected the cross-talk between the steroid hormone reception and signal transduction.

## Conflict of interest

Authors declare no conflict of interests.
